# A comparison of indoor and outdoor calf housing systems using automated and manual feeding methods and their effect on calf health, behavior, growth, and labor

**DOI:** 10.1093/jas/skac079

**Published:** 2022-03-15

**Authors:** Alison M Sinnott, Eddie A M Bokkers, John Paul Murphy, Emer Kennedy

**Affiliations:** 1 Teagasc, Animal and Grassland Research and Innovation Centre, Moorepark, Fermoy, Co. Cork, Ireland; 2 Animal Production Systems group, Wageningen University & Research, P.O. Box 338, 6700 AH Wageningen, The Netherlands

**Keywords:** animal welfare, dairy calves, feeding systems, housing systems, labor efficiency

## Abstract

Housing and feeding are integral to calf rearing, and must meet calf needs while remaining functional for the farmer. This study compared health, behavior, growth, and labor requirements of calves housed in groups indoors and fed via an automatic or manual milk feeding system compared to calves manually fed in individual or group hutches outdoors. Seventy-six (49 Holstein Friesian [**HF**] and 27 HF × Jersey) dairy heifer calves were balanced for birth weight (35.2 ± 4.95 kg), birth date (1 February ± 7.2 d) and breed. The experiment was a randomized block design with four treatments; 1) indoor group housing with automated feeding (IN_AUTO; 12 calves per pen), 2) indoor group housing with manual feeding (IN_MAN; 12 calves per pen), 3) outdoor group hutch with manual feeding (OUT_G_MAN; 8 calves per pen), and 4) outdoor individual hutch with manual feeding (OUT_I_MAN; 6 calves: 1 per pen). Calves in OUT_treatments moved outdoors at 18 d (± 5.9 d). Each treatment was replicated once. Milk allowance increased gradually from 6 to 8 L/day (15% reconstitution rate) with ad libitum fresh water, concentrates, and hay offered from 3 d old. Gradual weaning occurred at 8 wk old. Measurements were divided into period 1; before movement outdoors, and period 2; after movement outdoors. Health was similar among treatments, regardless of period, with the most frequent score being zero (i.e., healthy). Summarized, standing and lying were observed 24.3% and 29.8%, respectively, in OUT_I_MAN calves, compared to 8.0% and 49.1%, for the other systems, which were similar. No difference in bodyweight (**BW**) existed between treatments, except at weaning where BW was lower for OUT_I_MAN (67.4 ± 2.84 kg) compared to IN_MAN (74.2 ± 2.01 kg), and day 102 where OUT_I_MAN (94.1 ± 2.85 kg) were lighter than IN_AUTO (101.1 ± 2.10 kg) (*P* = 0.047). Total labor input was greatest for OUT_I_MAN (00:02:02 per calf per day; hh:mm:ss) and least for IN_AUTO (00:00:21 per calf per day) (*P* < 0.001). The labor for feeding (00:00:29 per calf per day), feeding inspection (00:00:10 per calf per day), and cleaning equipment (00:00:30 per calf per day) was greatest for OUT_I_MAN. All calves showed good health and growth patterns. Differences in behavior expressed by calves in the OUT_I_MAN, compared to other treatments may indicate compromised welfare. Thus, although outdoor group hutches do not negatively impact calves, indoor housing, particularly using automated feeders, can improve labor efficiency.

## Lay Summary

In seasonal calving dairy systems, cows calve in a period of approximately 12 wk. Demand for calf accommodation and labor is high during this time. Outdoor housing structures, such as robust plastic calf hutches, may offer an alternative to permanent indoor facilities. In this study, we compared indoor housing systems using automated and manual feeding methods and outdoor calf housing systems using manual feeding methods, to examine their effect on calf health, behavior, growth, and labor. Moving calves to their respective outdoor system commenced at approximately 18 d. This reflected a housing system with limited indoor availability, where older calves would be moved outdoors (allowing young calves to remain indoors). The most labor-efficient method of rearing was group housing calves indoors feeding via automatic feeder, followed by group housing indoors feeding via manual feeders, outdoor in group hutches, and outdoor in individual hutches with manual feeding. Calves in all systems showed health and growth patterns consistent with positive development. Calf behavior in the individual hutches outdoor may indicate compromised well-being, compared to all other systems. Thus, although outdoor group hutches do not negatively impact the calf, indoor housing, particularly when using automatic feeders, can provide improved labor efficiency.

## Teaser

Indoor group housing using automatic feeders was the most labor-efficient system investigated. All calves displayed positive health and growth indicators; however, behavioral patterns of calves housed individually outdoors may suggest compromised well-being.

## Implications

This study compared indoor housing using automated and manual feeding methods and outdoor calf hutches using manual feeding methods, tracking their effect on calf health, behavior, growth, and labor. Regardless of housing and feeding system, only minor health issues were encountered and growth was good. Behavioral indicators showed that movement from an indoor group-housing system, to individual outdoor hutches may negatively impact calf well-being. In addition, indoor group housing, particularly those using automatic feeders, was the most labor-efficient calf-rearing system. This study provides research-based evidence to aid in decision-making surrounding housing and management practices to improve calf health, growth, and welfare.

## Introduction

Quotas previously placed on European milk production were abolished in 2015 and caused a change in dynamic of the dairy industry within the member states. Herd sizes decreased in some countries (e.g., Greece) while in others, such as Ireland and Germany, they increased (Eurostat, 2018; ICBF, 2019). Extra facilities and housing for expanding dairy herds have been built, but calves are still frequently housed in unsuitable existing farm facilities (O’Brien et al., 2007; Marcé et al., 2010), suggesting investment in housing specifically built for the purpose of calf rearing has not been prioritized.

Although there are numerous ways calves are housed, the most common types of calf accommodation include outdoor individual and paired hutches, as well as indoor individual and group housing. In Ireland, indoor group housing is the predominant type of calf accommodation (Marcé et al., 2010), with a number of farms using individual pens indoors after birth, grouping at 7 d old (Teagasc, 2017). In addition, compact calving targeting a 6-wk calving rate, due to its association with reproductive efficiency, is applied in Ireland (Shalloo et al., 2014). This further increases requirements for calf accommodation, particularly around peak calving, due to a large number of calves being born in a short time frame. In contrast, year-round calving systems use calf houses throughout the year and require less accommodation. Outdoor housing structures, such as heavy gauge plastic calf hutches, may offer a potential alternative to permanent indoor facilities (Marcé et al., 2010), particularly when required for relatively short periods of time in seasonal calving systems. However, the impact of outdoor hutches on both calf and farmer in such a system requires examination, in terms of calf health, behavior, growth, and labor efficiency.

Housing environment and management practices have an influence on calf health and welfare. Calf housing should facilitate a comfortable environment, minimize the requirement for veterinary assistance and labor, and contribute to a low morbidity and mortality (Lorenz et al., 2011). Rearing calves outdoors, compared to indoor environments, is acknowledged as advantageous in terms of calf health (Lorenz et al., 2011; Wójcik et al., 2013), weight gain and solid feed intakes (Wójcik et al., 2013). However, Jorgenson et al. (1970) and Kung et al. (1997) found no difference between indoor and outdoor preweaning growth rates. Whether outdoors or indoors, individual housing has also been linked to improved calf health (Marcé et al., 2010) but may lead to abnormal oral behaviors such as tongue rolling (Lv et al., 2021), excessive oral manipulation of objects and body parts (Bokkers and Koene, 2001). Conversely, paired and group housing encourages social development, particularly in developing feeding behaviors such as learning to select and eat appropriate foods (Costa et al., 2016; Mahendran et al., 2021), leading to less fearful calves than those individually reared (Costa et al., 2016) particularly in relation to novel foods (Whalin et al., 2018), but, it can result in behavioral issues such as cross-sucking of other calves (Jensen, 2003). Furthermore, previous research has reported increased competition surrounding access to milk with group-rearing calves (Miller-Cushon et al., 2014). Individual housing eliminates this, which may further improve calf growth. It should be recognized that differences in feeding and management practices exist between different studies when considering findings related to calf housing, health, behavior, and growth.

Previous research found no differences in labor between indoor and outdoor individual pens fed manually (assumption of feeding system based on the time period of study [1970]; 4 d postcalving until weaning at 3, 5, and 7 wk) (Jorgenson et al., 1970). The most recent research into the labor associated with hutches found indoor individual hutches feeding calves manually twice a day were more labor-intensive than grouping calves indoors with computerized feeders (from 4 d postcalving until weaning at 7 wk) (Kung et al., 1997). They suggest that grouping is more labor-efficient than individually rearing calves. This may be due to feeding method rather than housing system. This is confirmed by Sinnott et al. (2021) who showed large differences exist in the labor associated with manual feeding twice a day and automatic feeding systems for indoor group-reared calves, indicating labor efficiencies may be closely related to feeding systems rather than housing system.

Housing and feeding are integral to successful calf-rearing operations and must remain functional for the farmer, while meeting the calf’s needs. The overall aim of this study was to compare health, behavior, growth, and labor requirements of calves housed in groups indoors and fed via an automatic or manual milk feeding system compared to calves manually fed milk in individual or group hutches outdoors. The primary outcome was to investigate how housing and feeding system influence calf weight gain, and the secondary outcomes included the effects of these systems on calf health, behavior, and labor. The study hypothesis was that calf health, behavior, growth, and labor is affected by the housing and feeding system they are reared in: calves in outdoor systems would have improved health with behavioral profiles displaying increased abnormal behaviors when housed in individual hutches, whereas indoor systems would be more labor-efficient (particularly those using automated feeders).

## Materials and Methods

Experiments were undertaken, and animals cared for, in accordance with the European Union (Protection of Animals Used for Scientific Purposes) Regulations 2012 (S.I. No. 543 of 2012). Ethical approval to undertake the study was approved by the Teagasc Animal Ethics Committee. The study was conducted from January 29 to April 13, 2020 at Teagasc Moorepark Research Farm, County Cork, Ireland.

### Animals and experimental treatments

The study population consisted of 76 dairy heifer calves: 49 Holstein Friesian (**HF**) and 27 HF × Jersey. Calves were balanced and blocked into eight groups (two groups per treatment) based on birth weight (35.2 ± 4.95 kg), birth date (February 1 ± 7.2 d), and breed. There was no more than a 14-d difference in date of birth between calves within each group. The experiment was a randomized block design with four treatments: 1) indoor group housing with automated feeding (IN_AUTO; 12 calves per pen), 2) indoor group housing with manual feeding (IN_MAN; 12 calves per pen), 3) outdoor group hutch with manual feeding (OUT_G_MAN; 8 calves per pen), and 4) outdoor individual hutch with manual feeding (OUT_I_MAN; 6 pens). Each treatment was replicated once. Calves were assigned to treatments within approximately 3 d following birth. The experimental unit within this study was individual calf. All calves assigned to treatments in this study completed the study protocol and data collected were analyzed for both primary (investigating how housing and feeding system influence calf weight at weaning), and the secondary outcomes (investigating the effects of these systems on calf health, behavior, and labor).

### Calf management and housing

**Figure 1.**
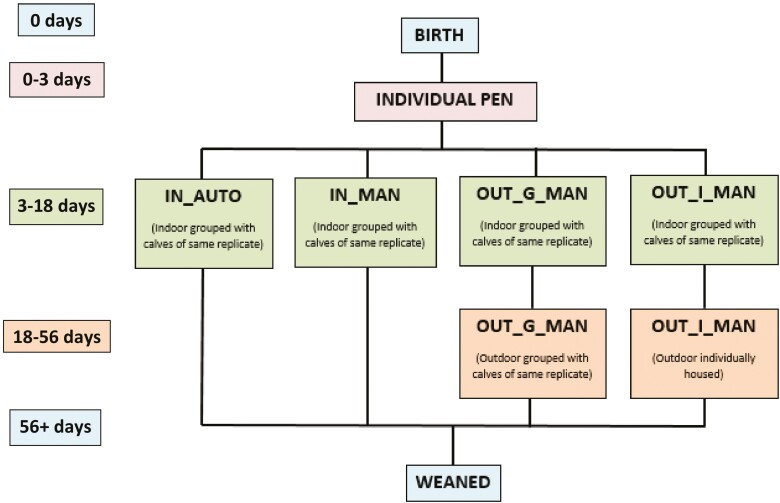
Schematic of calf flow through the study from birth (0 d) until weaning (56 d) for IN_AUTO (fed via automatic feeder from day 3 to 56), IN_MAN and OUT_G_MAN (both fed manually via compartmentalized multi-teat feeder from day 3 to 56), and OUT_I_MAN (fed manually via compartmentalized multi-teat feeder from day 3 to 18 and manually via individual feeder with teat from day 19 to 56).

A schematic of calf flow through treatment housing can be seen in Figure 1. All calves were immediately removed from their dam following calving as a biosecurity measure. Within the first hour of life, calves were weighed (TruTest XR 3000, Tru-test limited, Auckland, New Zealand) and placed in individual pens indoors (1.22 m × 0.82 m) with an overhanging infrared heat lamp. Calves received 8.5% of birth bodyweight (**BW**) of colostrum (Conneely et al., 2014) from a single cow (not specifically their own dam) via a teated bottle. Only colostrum that measured ≥22% Brix (Bielmann et al., 2010) was fed to calves. Calves received five feeds of transition milk (3 Liters per feed) after colostrum feeding, which was pooled from second milking postcalving. Feeding occurred at 8:00 and 16:00 with calves receiving the first feed of transition milk at the next feeding time postcolostrum consumption, and twice per day thereafter until treatment allocation and movement into an indoor group pen.

All calves were moved to an indoor group pen at approximately 3 d old. Calves of the same replicate and treatment were grouped together. Calves assigned to the automated feeding system were fed via automatic feeder (Volac Förster Technik Vario, Germany) and calves assigned to manual feeding systems were fed manually with a compartmentalized teat feeder (Milkbar, New Zealand; regardless of whether they were to be grouped or individually housed at a later stage in the study).

After the first indoor period, at an average 18.4 (± 5.96 d) d old, calves in the OUT_G_MAN and OUT_I_MAN treatment groups were moved to their respective housing outdoors. This reflected a housing situation whereby older calves are moved outdoors due to restricted indoor housing availability, allowing younger calves to remain indoors. In this period, OUT_I_MAN moved from a group to an individual housing system. Treatment groups were located adjacent to one another both indoors (IN_AUTO and IN_MAN) and outdoors (OUT_G_MAN and OUT_I_MAN). Visual and auditory cues could be exchanged between all calves housed outdoors and between all calves housed indoors. No physical contact was possible between treatment groups, with the only exception being calves in the OUT_I_MAN, who could touch between pen structures as per EU (Council Directive 2008/119/EC) and Irish welfare regulations (Planning and Development Regulations, 2001, S124). The treatments housed indoors (i.e., IN_AUTO and IN_MAN) remained in the same group pen throughout the study. Calves in outdoor treatments (i.e., OUT_I_MAN and OUT_G_MAN) in replicate 1 were moved outdoors on week 3 of experiment and calves in replicate 2 were moved to their outdoor housing on week 4 of experiment.

#### Indoor housing

The IN_AUTO and IN_MAN treatments were located in a calf-rearing shed. Group pens provided a space allowance of 2.9 m^2^ per calf, and had a concrete feeding area and a separate straw-bedded area (1.8 m^2^ per calf; 15 cm straw depth). Bedding was fully removed weekly, the area disinfected and re-bedded with fresh straw. Straw was replenished midway through the week ensuring a clean, dry bed was consistently available. Solid dividers between pens prevented physical contact between groups.

#### Outdoor housing

Following a period of indoor housing (same housing parameters as IN_MAN and IN_AUTO described above), calves were moved to their respective outdoor hutches. Outdoor group and individual hutches were placed on a concrete surface and positioned north-facing, which protected the hutch entrance from the prevailing wind. Drainage channels collected and removed run-off, from bedding and feeding areas, to appropriate storage facilities. Group and individual hutches were straw-bedded; straw was replenished three times weekly; bedding was not removed from the hutches during the experiment. The outdoor concrete feeding area was cleaned three times weekly.

Outdoor group hutches were constructed according to manufacturing guidelines (Super Hutch, JFC AGRI, Galway, Ireland) providing a space allowance (bedded and yard area) of 1.4 m^2^ per calf. Each pen contained an outdoor concrete feeding area enclosed by gates (JFC AGRI, Galway, Ireland), and a covered bedded area (molded heavy gauge plastic; 0.9 m^2^ per calf). Outdoor individual hutches had a space allowance of 4.9 m^2^ per calf, including an outdoor concrete feeding area enclosed by gates (JFC AGRI, Galway, Ireland) and a covered bedded area (2.6 m^2^ per calf).

### Calf feeding plan

All calves were offered 26% crude protein milk replacer (Volac Heiferlac; Volac, Church St., Portaliff Glebe, Killashandra, Co. Cavan; 26% crude protein, 16% crude oils and fats, 7% crude ash) reconstituted at 15%, using warm water which was fed immediately. From 2 wk old, milk allocation was adjusted every 0.5 wk, based on the age of the calf (Supplementary File 1). The total milk powder allowance for each calf (regardless of treatment) throughout the experiment was 46.4 kg. Calves remained on 6 L/day until 2 wk old. Daily milk allowance increased on an individual calf basis from 6 to 8 L/day gradually, in 0.5-Liter increments every 0.5 wk. Once a calf was consuming 8 L/day for 1 wk (week 4 to 5), milk allowance was reduced by 1-Liter increments every 0.5 wk until weaning at 8 wk old (56 d).

Concentrates (20% crude protein; composed of barley, soya meal, sugar beet pulp, distillers grains, rape seed meal, maize; Sweet Start Calf Starter Pencils, Southern Milling, Cork, Ireland), water and hay were offered ad libitum from 3 d old.

### Feeding system

Two feeding systems were used; automatic and manual feeding systems.

#### Automatic feeding system

For the IN_AUTO treatment, group pens were fitted with one automatic milk and concentrate feeding station (one automatic milk feeding unit supplied four feeding stations within separate group pens). The calf to teat ratio was 12 calves to one feeding station. According to individual calf feeding plans, the automatic feeder mixed and distributed milk. Each calf was allocated four feeds spaced evenly throughout the day (24 h) from 4 d old; access to the feeder was granted until the allocation for that feed was fully consumed, after this calves waited for 4 h until access was allowed for the next allocation. The automatic concentrate feeder provided individual ad libitum access, dispensing 200 g of concentrate as required to minimize wastage and increase distribution accuracy.

#### Manual calf feeding system

Manual calf feeding systems used for IN_MAN and OUT_G_MAN treatments consisted of plastic compartmentalized teat feeders. The OUT_I_MAN were fed using individual bucket teat feeders (2 Gallon Green Bucket, JFC AGRI, Galway, Ireland). Feeders were not shared between treatments and the calf to teat ratio was one calf to one teat. Calves were offered milk twice daily in two equal feeds (08:00 and 16:00). Concentrates were offered on an ad libitum basis from 3 d old with one open concentrate feeder (McInnes Manufacturing, New Zealand) per group.

### Measurements

#### Bodyweight

BW was measured at birth, weekly thereafter until weaning, and fortnightly postweaning, using the weighing scale described above. Weighing ceased temporarily once calves were weaned, until calves were approximately 102 d old due to logistical complications resulting from COVID-19 regulations.

#### Health

Individual health scores were assigned twice weekly from 3 d old to weaning (56 d old), in the late morning/afternoon (11:00 to 12:00). Measurements were carried out by a single experienced observer, with training previously validated via trainer interobserver reliability. Calf health scoring criteria and methodology outlined by Sinnott et al. (2021) was used (Supplementary File 2). In short, each calf was assessed individually and awarded a health score (4-point scale; 0 = excellent, no issues; 3 = severe issues; modified Madison Wisconsin health scoring system), for health factors twice weekly including: demeanor, nasal discharge, ocular discharge, ear position, attitude, coughing, dehydration, mobility, and hindquarter cleanliness (fecal cleanliness).

It is important to note that individual health inspections are independent of routine daily health checks carried out by the farmer (seen later as part of the labor evaluation). The labor associated with health scoring twice weekly was not recorded because it was an in-depth and time-consuming process conducted by a researcher, nonreflective of inspections taking place on commercial farms.

#### Behavior

Behavioral observations were undertaken weekly during the preweaning period (from day 3 until week 7 of replicate 1 and week 6 of replicate 2 due to COVID-19 regulations). Observations were carried out by a single experienced observer, with training in calf behavior recognition prior to commencement of this study. Prior to data collection, behavioral patterns were defined referring to the calf ethogram reported by Barry et al. (2019) (Supplementary File 3). Behaviors included: standing, lying, walking, defecating/urinating, drinking water, eating, scratching/rubbing/stretching, tongue playing/rolling, urine drinking/oral manipulate prepuce/cross-sucking, orally manipulating pen structure, play behavior/mounting/head butting. Data were collected four times per day (10:30, 12:00, 14:30, and 16:30) using methodology outlined by Sinnott et al. (2021). In brief, measurements were taken of each calf, within each treatment, using scan sampling at 1-min intervals for 15 min.

#### Labor

Labor evaluations were completed twice weekly from approximately 18 d of age (i.e., from the moment calves in OUT_treatments were moved out of the indoor pens), until week 7 for animals in replicate 1 and week 6 for animals in replicate 2 (due to COVID-19 regulations) during the trial period. Individual tasks associated with calf rearing described by Sinnott et al. (2021) were assessed including: feed preparation, feeding, cleaning feeding equipment, cleaning pen, and health checks. Measurements were repeated in the morning and evening and timed using a stopwatch. The sum (total hh:mm:ss) of each labor task quantified per day was divided by the number of calves in the pen or hutch that day. Specific descriptions of labor cues for each respective task are included in Supplementary File 4. All figures related to labor are reported in the following format: hh:mm:ss.

Transportation was included to record the additional time required to move milk and feeding equipment from the calf-rearing shed to the outdoor hutches via a Gator Utility Vehicle (John Deere, Illinois, USA). This time requirement included the moving, loading and unloading of milk and feeding equipment from the preparation area to the hutches and the return of all equipment for cleaning. Feeding inspection was amended from the protocol by Sinnott et al. (2021) and redefined as the time required to confirm all manually fed calves were drinking milk during feeding time, and the time required to inspect the handheld device to ensure that all calves were consuming milk on the automatic feeding system. Feeding inspection and health checks were carried out simultaneously for manually fed calves, while for automated-fed calves they were carried out at different times. Health checks consisted of an evaluation of each calf to ensure there were no obvious signs of illness, they had a good demeanor, could stand and walk, and that manually fed calves had an interest in consuming their milk allocation.

#### Temperature and relative humidity

Temperature and relative humidity were measured every 10 min during the study using data loggers (Tinytag TGP 4017 Temperature Data Logger; Gemini Data Loggers, West Sussex, United Kingdom). Loggers were positioned inside the lying area, out of calf reach (1.5 m from ground level). A weather station located <1 km from the experimental site recorded external weather conditions (Met Eireann, Fermoy, Cork, Ireland).

### Data editing

Measurements were conducted on calves from 3 d old. This allowed the establishment of baseline measurements such as calf health, behavior, and growth. For this reason, data will be divided into two periods hereafter; period 1 refers to the time all calves were housed indoors in group pens (approximately 3 to 18 d old) and period 2 refers to the time after when two treatments (OUT_G_MAN and OUT_I_MAN) moved outdoors to their respective treatments while IN_AUTO and IN_MAN remained indoors, until weaning (approximately 18 to 56 d old).

Due to complications resulting from COVID-19 pandemic restrictions, the labor and behaviors associated with replicate 1 are reported until week 7 and week 6 for replicate 2. It was also not possible to record weights from 60 d old to 102 d old (postweaning period) for this reason. Regarding labor, pen cleaning for the OUT_G_MAN and OUT_I_MAN treatments occurred too infrequently to be accurately included in statistical analysis. As outdoor hutches were previously perceived as labor-intensive (Lundell, 2015) particularly for cleaning, it was important to include this task to compare treatments in an unbiased way. Consequently, pen-cleaning figures are reported in text as raw averages for OUT_G_MAN and OUT_I_MAN treatments; furthermore, figures for pen cleaning were omitted from the total labor input for all treatments in Table 1. As all calves in manual feeding systems were housed in similar conditions for period 1, labor was not recorded for calves until located in their respective outdoor treatments. Due to incomparable calf numbers across treatment pens, figures referring to labor are on a per calf basis rather than per pen. Additionally, due to incomparable behaviors between replicates for week 7 (due to COVID-19 complications), behavioral data are reported until week 6 across all treatments and replicates.

**Table 1.** Mean labor input per calf per day (± SEM; hh:mm:ss; mean across treatments) and mean labor input per pen per day (± SEM; hh:mm:ss) for tasks associated with rearing calves for indoor automatic (IN_AUTO), indoor manual (IN_MAN), outdoor group manual (OUT_G_MAN), and outdoor individual manual (OUT_I_MAN) feeding systems from day 18 until weaning at 56 d

(hh:mm:ss)	Indoor		Outdoor		SEM	*P*-value
	IN_AUTO	IN_MAN	OUT_G_MAN	OUT_I_MAN		
Per calf per day						
Total time	00:00:21a	00:00:55b	00:01:27c	00:02:02d	00:00:07	0.001
Feed preparation	00:00:03a	00:00:25c	00:00:29b	00:00:29b	00:00:01	0.001
Transport	00:00:00a	00:00:00a	00:00:23b	00:00:24b	00:00:01	0.001
Feeding	00:00:00a	00:00:08d	00:00:06b	00:00:29c	00:00:01	0.001
Feeding inspection	00:00:06a	00:00:06a	00:00:07a	00:00:10b	00:00:01	0.001
Clean equipment	00:00:02a	00:00:16b	00:00:22c	00:00:30d	00:00:02	0.001
Health inspection	00:00:11a	00:00:00b	00:00:00b	00:00:00b	00:00:01	0.001
Per pen per day						
Total time	00:03:55a	00:10:40bc	00:09:53c	00:20:20d	00:00:28	0.001
Feed preparation	00:00:33a	00:04:56c	00:03:22b	00:04:44c	00:00:13	0.001
Transport	00:00:00a	00:00:00a	00:02:33c	00:03:49b	00:00:04	0.001
Feeding	00:00:00a	00:01:32d	00:00:41b	00:05:05c	00:00:07	0.001
Feeding inspection	00:01:06a	00:01:05a	00:00:47b	00:01:43c	00:00:07	0.002
Clean equipment	00:00:25a	00:03:07b	00:02:30b	00:04:59c	00:00:18	0.001
Health inspection	00:01:52a	00:00:00b	00:00:00b	00:00:00b	00:00:07	0.001

Different letters within row indicate statistical difference *P* ≤ 0.05.

To analyze health scores binary data were created, individually for each health factor, whereby four scores for each health factor (0, 1, 2, 3) were recategorized into two categories; category 1 are calves that scored zero and category 2 are calves that scored anything above zero (i.e., indication of health issue). An additional summary parameter was included which divided time (weeks) into two categories; period 1 and period 2. Environmental temperatures from TinyTag data loggers were included in the health data. Average figures were calculated for temperatures recorded between data collection days and divided into two categories; category 1 are temperatures which fall below the thermoneutral zone (**TNZ**) based on age (i.e., lower critical temperature [**LCT**]; ≤10 °C; Davis and Drackley, 1998) and category 2, where temperatures fall within the TNZ of the calf (≥11 to 24 °C). Orthogonal contrasts were added to compare effects of temperature, period, and location on calf health: LCT vs. TNZ, period 1 vs. period 2, and indoor vs. outdoor. Contrasts, related to location, were carried out to evaluate the health of calves housed indoors and outdoors.

Although treatment replicates were separate from one another (except OUT_I_MAN, whereby visual and tactile stimulation was possible), we acknowledge that an in-pen influence exists for calf behavior. Consequently, all results pertaining to calf behavior in period 1 and 2 are reported in a descriptive capacity. The 17 potential behavioral outcomes were condensed into nine categories (Table 2); standing, lying, rumination, feeding-related behaviors, comforting, abnormal behaviors, play, tactile social interaction with another calf, and other behaviors. All categories were mutually exclusive (e.g., postural behaviors such as standing and lying were superseded by other behaviors, such as rumination, when other behaviors were observed). Grouping allowed for the analysis of behaviors that occurred infrequently (such as behaviors in the abnormal and feeding categories), which may have otherwise went undocumented statistically. An average figure was calculated from temperatures recorded by the TinyTag data logger during the specific date and time that observations were made and divided into two categories (mentioned above) and included as a variable in the data set. Orthogonal contrasts were used to compare the effects of temperature, period, and location on behavior, including comparisons of the following: LCT vs. TNZ, period 1 vs. 2, and indoor vs. outdoor.

**Table 2.** Nine mutually exclusive behavioral categories based on various observed behaviors, used for behavioral analysis

Category	Observed behaviors^1^
Standing	Standing (only)
Lying	Lying (only)
Rumination	Rumination while standing Rumination while lying
Feeding	Drinking milk Drinking water Eating concentrate Eating forage
Comforting	Grooming Scratching/rubbing/stretching
Abnormal	Tongue playing Oral manipulation of the prepuce Oral manipulation of the pen (excessive: continuous and repeated to high frequencies) Cross-suckling
Play	Galloping Bucking/hind leg kicking Body rotations/twisting
Tactile social interaction	Licking/allo-grooming another calf Touching another calf Nuzzling another calf
Other	All other behaviors (e.g., walking, defecating/urinating)

Detailed descriptions of the observed behaviors can be found in the study by Sinnott et al. (2021).

BW data were organized according to calf age rather than calendar date. Calves were weighed on a fixed day every week. For accuracy data were corrected, allowing for age comparisons among calves. The calf-specific average daily gain (**ADG**) on each weighing date was calculated based on the difference between weighing dates and weight values. ADG was divided into pre- and postweaning for each calf individually. The pre- and postweaning periods were 59 and 93 d, respectively. As all calves were housed indoors for a period, each ADG recorded for this period was summarized and averaged for a common indoor ADG figure. Orthogonal contrasts (indoor vs. outdoor) were carried out to determine effects on calf weight gain.

Average figures were calculated from temperature and humidity data, related to indoor and outdoor environments, based on 144 daily environmental measurements. Minimum and maximum temperatures were also noted. The LCT threshold was used as a comparison against average figures to determine whether calves were within their daily TNZ. Average environmental (housing lying area) and atmospheric temperatures were compared to determine differences.

### Statistical analysis

Sample size was calculated based on our primary outcome (namely weaning weight) using existing results from a previous body of work (Sinnott et al., 2021) with 95% confidence interval (**CI**) and 80% power. This sample size would allow for detection of a 2.5 kg difference in weaning weight. Statistical analyses were conducted using SAS (Version 9.4, SAS Institute Inc., 2002). Linear mixed models (PROC MIXED) were used to evaluate whether treatment affected labor input and calf growth (BW and ADG) when rearing calves; multiple comparisons and their interactions were assessed using the PDIFF option in the least square means statement with Tukey adjustment. Dependent variables used followed a normal distribution pattern. Significant associations were confirmed at *P* ≤ 0.05. Categorical variables were treatment, calf number, and replicate. Related to labor, fixed effects were treatment, replicate, and week-of-treatment. The time parameter, week-of-treatment, refers to the weeks after all calves are exposed to their treatment, which is after approximately 18 d. Week-of-treatment number 1 is therefore the first week after approximately 18 d. For data related to BW and ADG, week-of-treatment was not included as a fixed effect; instead birth weight, date of birth, period, and breed were included. An additional covariate was included in the model accounting for the difference in age between individual calves at each weighing date. Time of measurement was the repeated measure used in the model.

The frequency procedure (PROC FREQ) was used to describe the non-normal distribution of categorical variables related to health scoring. Data were sorted according to treatment. Associations between the independent variables, treatment, location, period, temperature, and replicate, on fecal health scores were completed using the logistic regression procedure (PROC LOGISTIC; binary distribution with link logit). The following reference categories (odds ratio [**OR**] = 1) were used: the IN_AUTO treatment, indoor location, thermoneutral temperature (11 to 16 °C) and replicate 1. The model is described using ORs, with a 95% CI.

The frequency procedure (PROC FREQ) also described the non-normal distribution of behavior related to treatment. Data were sorted according to treatment and period. Logistic regression (PROC LOGISTIC) was used to determine the associations between the independent variables: location, temperature, and replicate (each of which were dichotomous variables), on calf behavior (nine behavioral categories with binary outcomes). The indoor environment, thermoneutral temperature (11 to 16 °C), and replicate 1 were designated as the reference categories. The dependent variable “standing” was designated as the reference category for behavior analysis, meaning each behavior was compared to the standing variable. The model is described using ORs, with a 95% CI.

## Results

### BW and ADG

There were no differences in BW for period 1 (14 and 21 d; 40.6 ± 2.38 kg and 44.9 ± 2.35 kg, respectively). Treatment did not have an effect on BW in period 2 at 36 (55.5 ± 2.36 kg), 43 (60.3 ± 2.35 kg), postweaning at 123 (111.7 ± 2.36 kg), or 156 (136.9 ± 2.36 kg) d of age. At weaning, BW of OUT_I_MAN calves was lower than IN_MAN calves (67.4 ± 2.84 kg and 74.2 ± 2.01 kg, respectively; *P* = 0.05). At 102 d, there was a difference in BW between OUT_I_MAN and IN_AUTO calves (94.1 ± 2.85 kg and 101.1 ± 2.10 kg; *P* = 0.047; all other treatments were similar).

No differences were found in the ADG for period 1 (0.52 ± 0.03 kg/day). There were no differences in ADG between treatments at 36, 43, 123, or 156 d of age. The ADG during the weaning period (49 to 56 d) was lower for OUT_I_MAN calves compared to IN_AUTO, IN_MAN, and OUT_G_MAN calves (1.3 ± 0.05, 2.5 ± 0.03, 2.2 ± 0.03, 2.9 ± 0.04 kg/day, respectively; *P* < 0.01). Location of calf housing (indoor or outdoor) did not influence calf BW (*P* = 0.737) or ADG (*P* = 0.998) throughout the study.

### Health

The majority of calves in each treatment were scored as 0, i.e., healthy, for each health score (Table 3). More calves had poor fecal cleanliness than any other health score. Treatment, period, location of the calves (indoor or outdoor), and ambient temperature did not influence fecal health scores (*P* = 0.092, *P* = 0.263, *P* = 0.262, and *P* = 0.964, respectively).

**Table 3.** Distribution frequencies (%; *N* = total number of observations) of health scores^1^ for indoor automatic (IN_AUTO; *N* = 313), indoor manual (IN_MAN; *N* = 340), outdoor group manual (OUT_G_MAN; *N* = 221), and outdoor individual manual (OUT_I_MAN; *N* = 162) feeding systems

Health factor	Health score per feeding system (%)							
	IN_AUTO (*N* = 313)		IN_MAN (*N* = 340)		OUT_G_MAN (*N* = 221)		OUT_I_MAN (*N* = 162)	
	0)	(≥1) )	0)	(≥1) )	0)	(≥1) )	0)	(≥1) )
Demeanor	100	0.0	100	0.0	100	0.0	100	0.0
Ear position	100	0.0	99.7	0.3	100	0.0	100	0.0
Eye secretion	100	0.0	100	0.0	99.5	0.5	100	0.0
Nasal discharge	100	0.0	100	0.0	99.5	0.5	100	0.0
Cough	100	0.0	100	0.0	100	0.0	100	0.0
Dehydration	100	0.0	100	0.0	100	0.0	100	0.0
Mobility	100	0.0	100	0.0	100	0.0	100	0.0
Interest	100	0.0	100	0.0	100	0.0	100	0.0
Fecal cleanliness	97.8	2.2	95.3	4.7	94.6	5.4	94.4	5.6

Health parameters assessed using a 4-point scale which was dichotomized and a score of 0 indicated absence the symptom/sign, and a score of 1, 2, or 3 indicated the presence an abnormal symptom.

### Behavior

**Figure 2.**
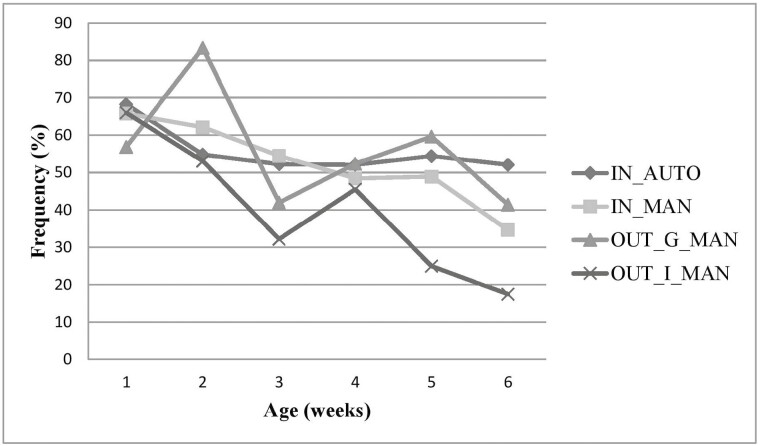
Frequency of lying behaviors (% based on the number of scans) expressed by age (in weeks) for indoor automatic (IN_AUTO), indoor manual (IN_MAN), outdoor group manual (OUT_G_MAN), and outdoor individual manual (OUT_I_MAN) feeding systems.

**Figure 3.**
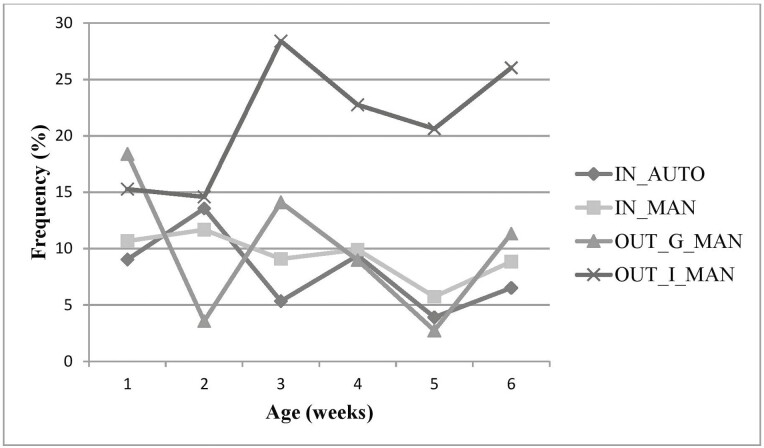
Frequency of standing behaviors (% based on number of scans) expressed by age (in weeks) for indoor automatic (IN_AUTO), indoor manual (IN_MAN), outdoor group manual (OUT_G_MAN), and outdoor individual manual (OUT_I_MAN) feeding systems.

Results pertaining to behavior are reported as a percentage based on the number of scans (*n*) relative to the total number of observations (*N*) in period 1 and 2. The total number of observations in period 1 and 2 for each treatment were as follows: IN_AUTO *N* = 1,325 and 2,307, IN_MAN *N* = 1,296 and 2,208, OUT_G_MAN *N* = 624 and 1,521, and OUT_I_MAN *N* = 336 and 1,281, respectively. Frequency analysis of behavior indicates lying was the most common behavior exhibited among treatments in both period 1 and 2 (Table 4). In period 2, calves in the OUT_I_MAN expressed lying and tactile social behaviors on average 29.8% and 0.9% of the total observation time, whereas IN_AUTO, IN_MAN, and OUT_G_MAN calves expressed these behaviors in a higher proportion (average lying and social tactile for the three treatments were 49.1% and 5.1%, respectively). Furthermore, standing and comforting behaviors were observed more frequently, in the OUT_I_MAN (24.3% and 9.4% of the total time, respectively) than IN_AUTO, IN_MAN, and OUT_G_MAN, which were similar (approximately 8.0% and 3.4% of the total time, respectively). A decreased level of lying behaviors was seen in OUT_I_MAN calves and despite an overall decrease over time, remained lower than all other treatments (except week 4; Figure 2). A similar, but reversed pattern was seen in standing behavior across the preweaning period among OUT_I_MAN calves (Figure 3). In period 2, the calves expressed feeding behaviors approximately 3.5% (OUT_G_MAN), 8.5% (IN_AUTO), 8.3% (IN_MAN), and 8.1% (OUT_I_MAN calves) of the total time, respectively.

**Table 4.** Frequency (% based on the number of scans; *N* = total number of observations) of behaviors exhibited by calves in period 1 (P1; day 3 to approximately 18) and period 2 (P2; day 19 to 56) in indoor automatic (IN_AUTO), indoor manual (IN_MAN), outdoor group manual (OUT_G_MAN), and outdoor individual manual (OUT_I_MAN) feeding systems

	IN_AUTO (%)		IN_MAN (%)		OUT_G_MAN (%)		OUT_I_MAN (%)	
	P1 (*N* = 1,325)	P2 (*N* = 2,307)	P1 (*N* = 1,296)	P2 (*N* = 2,208)	P1 (*N* = 624)	P2 (*N* = 1,521)	P1 (*N* = 336)	P2 (*N* = 1,281)
Standing	11.2	6.3	11.1	8.4	9.9	9.2	14.5	24.3
Lying	60.7	52.7	63.6	46.5	63.4	48.2	57.7	29.8
Rumination	3.4	21.9	2.9	21.7	9.7	25.8	3.5	19.1
General	2.0	1.9	4.0	2.5	2.7	1.6	3.2	3.2
Feed	8.6	8.5	7.3	8.3	2.0	3.5	5.2	8.1
Comfort	3.5	2.6	2.8	3.9	2.9	3.6	6.4	9.4
Abnormal	2.5	1.1	1.1	1.8	0.0	1.0	2.5	2.6
Play	2.7	1.3	3.8	1.3	0.9	1.1	1.3	2.7
Social	5.4	3.8	3.4	5.6	8.5	6.1	5.6	0.9

Experience of temperatures below the estimated LCT (≤10 °C) increased likelihoods of lying (OR = 1.81; CI = 1.59 to 2.06), rumination (OR = 2.34; CI = 2.00 to 2.73), and feeding (OR = 1.33; CI = 1.11 to 1.60). Whereas LCTs were associated with a decreased likelihood of oral pen manipulation (OR = 0.58; CI = 0.42 to 0.80), tactile social interactions (OR = 0.78; CI = 0.64 to 0.97), and playful (OR = 0.64; CI = 0.47 to 0.88) behaviors.

No incidences of tongue playing were recorded. Oral manipulation of the prepuce occurred twice, both of which were in succession by a single calf in the IN_MAN treatment, during week 4 of the experiment.

### Labor

Total labor input per calf per day, excluding cleaning, was different between all treatments (Table 1). The total labor input was greatest for OUT_I_MAN and least for IN_AUTO. Although not statistically analyzed, total labor input, including pen cleaning follows the same pattern and was greatest for OUT_I_MAN (00:03:08 per calf per day of measurement), followed by OUT_G_MAN (00:01:51 per calf per day of measurement), IN_MAN (00:01:01 per calf per day), and IN_AUTO (00:00:28 per calf per day). The labor input (per calf per day) for feeding, feeding inspection, and cleaning equipment was greater for OUT_I_MAN than any other treatment. The time taken to prepare milk for feeding, on a per calf basis, was greatest for OUT_G_MAN and OUT_I_MAN. Labor required for health inspection for the IN_AUTO was higher (+00:00:11 per calf per day) than any other treatment.

### Temperature and relative humidity

The average atmospheric temperature was 7.1 °C for the 53 d of treatment measurements, with a temperature range from −2.9 to 19.4 °C (consistent with long-term seasonal averages). For period 1, when all calves were housed indoors, the average ambient temperature was 8.1 °C, with calves experiencing temperatures outside of their TNZ 11 out of 14 d. Average ambient temperatures for indoor housing, and outside group and individual hutches for period 2 were 9.3 (range from 2.3 to 21.6 °C), 11.0 (range from −2.5 to 34.5 °C), and 9.5 °C (range from −1.3 to 32.8 °C), respectively. Average daily temperatures in period 2 showed calves housed indoors, in group, and individual hutches experienced temperatures outside of their TNZ for 36, 25, and 33 d, respectively.

The average indoor relative humidity when all calves were housed indoors in period 1 was 88.0%. The average relative humidity for the indoor housing, and inside the outdoor group and individual hutches in period 2 were 82.0%, 80.0%, and 81.2%, respectively. The lowest humidity recorded was 47.2% for the indoor environment, 26.2% for the group, and 22.2% for the individual hutches, all of which were in period 2. The maximum humidity of 100% was reached by all treatments periodically during the study. Average daily relative humidity experienced by calves in period 2 housed indoors, in group, and individual hutches was >90% for 8, 6, and 7 d, respectively.

## Discussion

### Behavior, weight, and temperature

Although behavioral data are reported in a descriptive capacity, this study is consistent with numerous others, indicating the most common behavior expressed by calves in early life is lying (see, for example, also Calvo-Lorenzo et al., 2016; Sinnott et al., 2021). A positive correlation between levels of rest and growth rates for growing cattle (Mogensen et al., 1997) highlights the importance of lying for the welfare of young growing animals. Previous studies have indicated that calves spend upwards of 50% of the day lying down in early life, typically decreasing over time (Webster et al., 1985; Chua et al., 2002; Hänninen et al., 2005). Although calves in all treatments displayed similar patterns to the above in early life (period 1), the OUT_I_MAN treatment expressed lying less than any other treatment, particularly after movement from indoors to outdoors. It is possible that deviations in behavior between period 1 and period 2 may be attributable to calves moving outdoors to their respective treatments, particularly with the movement from a group to an individual housing setting. It may be argued that calves were conditioned to stand in the presence of a human during behavioral observations due to positive feed reward associations (Krohn et al., 2001). However in light of this, it would be likely that all manual feeding systems would respond in a similar way, which was not the case. Additionally, in the case of outdoor hutches, conditioning in this study would likely be associated with feeding equipment and vehicles used to transport milk, meaning OUT_G_MAN would exhibit a similar profile for OUT_I_MAN, which also did not occur. With that being said, when calves were lying in their respective individual hutch, visual stimulation from other calves was not possible (unless calves were in the concrete pen outside the hutch). This may not have been favorable for calves, as short-term visual isolation from other calves is reportedly distressing (Bøe and Færevik, 2003). Calves may have been more alert or easily disturbed because, as herd animals, they lacked the security that grouping offers. In addition to this sentiment, it may also be possible that individually housed calves attempted to interact with a different species (humans), when the opportunity presented itself, in order to satisfy their social needs. Social tactile behaviors were also lower for calves in the OUT_I_MAN group; however, this decrease was expected because although some social contact was possible in the outdoor part of the hutch, it was limited substantially, compared to a group housing situation where social contact is readily available. It appears the shortfall of lying and social tactile behaviors is compensated for with an increase in standing and comforting behaviors (self-grooming, scratching, rubbing, and stretching), for the OUT_I_MAN calves, which were frequently carried out in the outer pen structure in view of other calves. This suggests that OUT_I_MAN calves have a high motivation for social interaction (Færevik et al., 2006). A notable increase in rumination was found from period 1 to period 2 across all systems, which is likely attributable to a correlation between increased age and rumen development facilitating this behavior (Swanson and Harris, 1958). Additionally, an increase in feed-related behaviors was also seen across manual feeding systems (consistently high in IN_AUTO relative to manual systems, likely due to the ability to consume milk during observation periods), which may also be attributable to rumen development promoting the digestion of feed, unrelated to milk. Although abnormal behavior was low among calves housed individually in this study, studies have shown that group housing reduces the incidence of abnormal behaviors such as object licking and increases the opportunity of social interactions compared to individual housing (Chua et al., 2002; Tapkı, 2006). Despite studies suggesting competition and social housing increases calf intakes (González et al., 2008; Miller-Cushon and DeVries, 2015), we expected that visual stimulation from other calves consuming concentrate would offset this and the elimination of feed competition associated with group housing (Miller-Cushon et al., 2014) would lead to better weight gains among individually housed calves. Although we did not study the effect this would have on feeding behavior specifically, the weight gain of individually housed calves did not reflect any competitive advantage compared to the other systems.

Studies have shown young calves are motivated to express individual and social play behavior when their primary needs and thermal comfort are met (Jensen et al., 1998). This is reflected in our findings which demonstrated that calves were less likely to express social and play behaviors when temperatures were outside their TNZ, also notable with the occurrence of pen manipulation increasing with temperature. This can be viewed as exploring the environment; however, when expressed extensively or in a stereotypic way it can be considered abnormal (Lauber et al., 2006). Feeding, rumination, and lying increased as environmental temperatures decreased. Calves may have responded to a LCT by modifying their behavior for thermoregulation and energy metabolism (Hänninen et al., 2003). On average, temperatures in the indoor environment and individual hutches were 2 °C greater than the atmospheric temperature, whereas group hutches were 4 °C greater. The higher average daily temperatures recorded in hutches, particularly group hutches, compared to indoor housing indicate hutches are effective in creating microclimates. However, outdoor-housed calves are more exposed to environmental conditions than indoor calves (Nordlund, 2008), due to one hutch side being permanently open which may lead to the structures heating and cooling quicker than indoor housing facilities. Therefore, it was expected that calves housed outdoors would have a greater difficulty adjusting when moving from indoors to outdoors. This would then necessitate the direction of a higher proportion of feed intake toward maintenance (Hepola et al., 2006), leading to poorer weight gain compared to their indoor counterparts. Although calf intake was not recorded in this study, weight did not reflect impairment of calf growth, due to the possible redirection of feed intake toward maintenance. The OUT_G_MAN calves displayed decreased feeding behaviors, but slightly elevated levels of rumination, suggesting that feeding behaviors may have been carried out at a different time to observations. Indoor housed calves weighed more at weaning, possibly due to gut fill as no differences were observed in ADG or calf weights postweaning. Thus, housing system, irrespective of location, did not appear to impact calf growth negatively.

### Labor and health

The most labor-efficient system was the IN_AUTO treatment, similar to that reported by Sinnott et al. (2021), followed by the IN_MAN treatment, suggesting indoor housing is more labor-efficient than outdoor. Transport of milk was a large contributor to the difference in labor between the two locations. Transport time is relative to the distance hutches are from the feed preparation and cleaning area and whether facilities to prepare milk exist in close proximity to the hutches. In this study, hutches were located <30 m from the feed preparation area. However, the breakdown of total labor input for each system indicates that if transportation is removed, the ranking of each system in terms of labor efficiency would remain unchanged. The OUT_I_MAN treatment was least efficient, suggesting that grouping calves is probably more labor-efficient than individual housing (a finding supported by Chua et al. 2002), particularly for outdoor individual housing in relation to feeding and feeding inspection. It is possible that greater labor efficiencies among all treatments may be seen if carried out on a larger scale, due to economies of scale.

Cleaning equipment was least labor-intensive for the IN_AUTO treatment, which is largely attributable to the automatic feeder self-cleaning twice daily. It must be noted that automatic feeders often require additional manual cleaning (in addition to the self-cleaning feature; e.g., cleaning teats and feeding unit) which incur additional labor requirements. In comparison, manual feeders require internal and external manual cleaning of each feeding compartment. The cleaning input for manual feeders used outdoors was greater than indoors. As removing equipment from the indoor environment may introduce potentially pathogen-harboring dirt to equipment from external environments, it was important to ensure that equipment was cleaned thoroughly. As outlined by Van Os et al. (2021), pathogens can be transported from one area of the farm to another in many ways including clothing, tools, and other items and additional care must be taken to eliminate contamination for calf health. Cleaning equipment is a labor-intensive yet necessary process to help protect calves against illness (Barry et al., 2019). Similar to Sinnott et al. (2021), although the study size was not designed to detect differences in health problems, the low rates of illness and no difference between treatments for health scores is perhaps in-part reflective of the high sanitization levels implemented for feeding equipment on the research farm where the present study was conducted, but this might not be the case on all commercial farms. It should be noted that additional cleaning measures (carried out, but not quantified in this present study) to aid in reducing/eliminating harmful bacteria are required and have labor implications associated with the practice. The benefit of these practices is evident (Dietrich, 2015); however, the labor implications of its execution are expected to be far less for automated systems due to the absence of physical labor required.

It was not possible to evaluate outdoor pen cleaning using statistical analysis, due to infrequent occurrence. However, outdoor housing is believed to benefit calf health (Lorenz et al., 2011), so it is possible that external weather conditions, as well as the slightly lower average relative humidity in the outdoor hutches, may have compensated for and led to an infrequent need to clean outdoor pens. This may benefit calf health, because high levels of humidity (>90%) have been linked to bacterial survivability in housing environments (Lago et al., 2006). Although average temperatures were higher in both outdoor housing systems compared to indoor housing, it remained within a calf’s upper TNZ threshold (24 °C; Davis and Drackley, 1998).

Automatic milk feeders provide an additional advantage over manual feeding systems by means of monitoring and recording calf intake and feeding behavior data, which can also be used to identify sick animals (Sutherland et al., 2018). Additionally, individual manual feeding also allows for the efficient quantification of calf intake without substantial human intervention. In a manual group feeding system, in order to quantify calf intake and receive the same information as above, a person would be required to stay at the feeder to ensure all calves consumed their full milk allocation, which has the potential to increase the labor demand for this task considerably (Sinnott et al., 2021). It must be noted, however, that automatic feeders require elevated labor input in the early preweaning period in relation to IT training to use such a system, as well as training calves to the feeder (Sinnott et al., 2021; Medrano-Galarza et al., 2018) and calibration of the feeding system itself.

### Practical implications

Indoor housing with automated milk feeders is a labor-efficient way of rearing calves, followed by manually feeding indoors. Health, growth, and behavioral indicators show that these systems do not appear to impact calves in a negative way in this circumstance. Indoor housing provides shelter from extreme weather for both calf and manager; however, there are high costs associated with building such a structure. Furthermore, automatic feeders are costly, requiring continued maintenance once installed, compared to costs associated with manual feeders. As automated feeding technology progresses, the further adaptation of these feeders to outdoor environments may change the labor efficiency of outdoor systems.

Outdoor group hutches were more labor-efficient than individual calf hutches. Transportation of milk and topping up of weather-soiled bedding proved inconvenient with both outdoor systems. Although hutches shelter calves, both calf and manager were more susceptible to prevailing weather compared to indoor housing. This means that in countries such as Ireland, operating primarily spring calving systems, weather will have a considerable influence on the suitability of hutches as housing. This raises the question that if weather conditions such as these persist or worsen, is outdoor housing viable? A concern echoed by producers, whereby the improved working conditions associated with indoor automated feeding and related housing proved more favorable than manual feeding systems, particularly those using outdoor housing (Medrano-Galarza et al., 2017).

Unlike automatic feeding systems, indoor and outdoor manual systems allowed human interaction with all calves at feeding time to determine a calf’s health status relative to milk consumption, which is beneficial for both the farmer in terms of the amalgamation of tasks and labor efficiency, and the calf in relation to health detection. On the contrary, automatic feeders provide additional information regarding calf milk consumption and other feeding behaviors, which can be used as health indicators, without the presence of a human, thus resulting in greater efficiencies in this area. Additionally, individual systems granted high levels of control at feeding time, which allowed calves to consume their full milk allocation.

Although expressed behaviors for IN_AUTO, IN_MAN, and OUT_G_MAN prove relatively similar, OUT_I_MAN calf behavior deviated for their counterparts significantly (potentially through movement from an indoor group-housing system). It is imperative to recognize that commercial farm practices that echo such management practices (moving calves to individual outdoor hutches due to lack of space for newborn calves) may have lasting negative impacts on a calf’s welfare particularly in the area of socialization.

## Conclusion

Our results showed that after 18 d of age, indoor automatic feeding systems were consistently more labor-efficient than indoor manual, outdoor group hutch, and individual hutch feeding systems. Health and growth patterns among all treatments were consistent with positive calf development. Differences in behavioral patterns expressed by calves from 18 to 56 d of age in the outdoor individual hutches compared to all other treatments may indicate compromised well-being through movement from an indoor group-housing system. Thus, although outdoor group hutches do not negatively impact the calf, indoor housing, particularly when using automated feeders, can provide improved labor efficiency.

## Supplementary Material

skac079_suppl_Supplementary_Files

## Data Availability

Available upon request.

## Abbreviations

ADG: average daily gain
BW: bodyweight
CI: confidence interval
HF: Holstein Friesian
LCT: lower critical temperature
OR: odds ratio
TNZ: thermoneutral zone

## References

[CIT0001] Barry, J., E. Kennedy, R. Sayers, I. J. M. De Boer, and E. A. M. Bokkers. 2019. Development of a welfare assessment protocol for dairy calves from birth through to weaning. Anim. Welf. 28:331–344. doi:10.3168/jds.2019-16815

[CIT0002] Bielmann, V., J. Gillan, N. R. Perkins, A. L. Skidmore, S. Godden, and K. E. Leslie. 2010. An evaluation of Brix refractometry instruments for measurement of colostrum quality in dairy cattle. J. Dairy Sci. 93:3713–3721. doi:10.3168/jds.2009-2943 20655440

[CIT0003] Bøe, K. E., and G. Færevik. 2003. Grouping and social preferences in calves, heifers and cows. Appl. Anim. Behav. Sci. 80(3):175–190. doi:10.1016/S0168-1591(02)00217-4

[CIT0004] Bokkers, E. A. M., and P. Koene. 2001. Activity, oral behaviour and slaughter data as welfare indicators in veal calves: a comparison of three housing systems. Appl. Anim. Behav. Sci. 75(1):1–15. doi:10.1016/S0168-1591(01)00175-7

[CIT0005] Calvo-Lorenzo, M. S., L. E. Hulbert, A. L. Fowler, A. Louie, L. J. Gershwin, K. E. Pinkerton, M. A. Ballou, K. C. Klasing, and F. M. Mitloehner. 2016. Wooden hutch space allowance influences male Holstein calf health, performance, daily lying time, and respiratory immunity. J. Dairy Sci. 99(6):4678–4692. doi:10.3168/jds.2016-10888 27016829

[CIT0006] Chua, B., E. Coenen, D. J. van, and D. M. Weary. 2002. Effects of pair versus individual housing on the behavior and performance of dairy calves. J. Dairy Sci. 85:360–364. doi:10.3168/jds.s0022-0302(02)74082-4 11913695

[CIT0007] Conneely, M., D. P. Berry, J. P. Murphy, I. Lorenz, M. L. Doherty, and E. Kennedy. 2014. Effect of feeding colostrum at different volumes and subsequent number of transition milk feeds on the serum immunoglobulin G concentration and health status of dairy calves. J. Dairy Sci. 97:6991–7000. doi:10.3168/jds.2013-7494 25200772

[CIT0008] Costa, J. H. C., M. A. G. von Keyserlingk, and D. M. Weary. 2016. Invited review: effects of group housing of dairy calves on behavior, cognition, performance, and health. J. Dairy Sci. 99:2453–2467. doi:10.3168/jds.2015-10144 26874423

[CIT0009] Davis, C. L., and J. K. Drackley. 1998. The development, nutrition, and management of the young calf. Iowa State University Press. Wiley-Blackwell.

[CIT0010] Dietrich, A. M. 2015. Management, sanitation, and accuracy of automated calf feeders [Masters dissertation]. Virginia Polytechnic Institute and State University.

[CIT0011] Eurostat. 2018. Small and large farms in the EU—statistics from the farm structure survey. [accessed September 18, 2020]. https://ec.europa.eu/eurostat/statistics-explained/index.php?title=Small_and_large_farms_in_the_EU_-_statistics_from_the_farm_structure_survey&oldid=406560.

[CIT0012] Færevik, G., M. B. Jensen, and K. E. Bøe. 2006. Dairy calves social preferences and the significance of a companion animal during separation from the group. Appl. Anim. Behav. Sci. 99(3–4):205–221. doi:10.1016/j.applanim.2005.10.012

[CIT0013] González, L. A., A. Ferret, X. Manteca, J. L. Ruíz-de-la-Torre, S. Calsamiglia, M. Devant, and A. Bach. 2008. Effect of the number of concentrate feeding places per pen on performance, behavior, and welfare indicators of Friesian calves during the first month after arrival at the feedlot. J. Anim. Sci. 86:419–431. doi:10.2527/jas.2007-0362 17940151

[CIT0014] Hänninen, L., A. M. De Passillé, and J. Rushen. 2005. The effect of flooring type and social grouping on the rest and growth of dairy calves. Acta Agri. Scand. A—Anim. Sci. 91(3–4):193–204. doi:10.1016/j.applanim.2004.10.003

[CIT0015] Hänninen, L., H. Hepola, J. Rushen, A. M. De Passille, P. Pursiainen, V. M. Tuure, L. Syrjälä-Qvist, M. Pyykkönen, and H. Saloniemi. 2003. Resting behaviour, growth and diarrhoea incidence rate of young dairy calves housed individually or in groups in warm or cold buildings. Acta Agri. Scand. A—Anim. Sci. 53(1):21–28. doi:10.1080/09064700310002008

[CIT0016] Hepola, H., L. Hänninen, P. Pursiainen, V. M. Tuure, L. Syrjälä-Qvist, M. Pyykkönen, and H. Saloniemi. 2006. Feed intake and oral behaviour of dairy calves housed individually or in groups in warm or cold buildings. Livest. Sci. 105(1–3):94–104. doi:10.1016/j.livsci.2006.04.033

[CIT0017] ICBF. 2019. Dairy cow population by county 2016–2019. https://www.icbf.com/wp/?p=14603#:~:text=Total%20dairy%20cow%20numbers%20for,1.6%25%20from%201%2C496%2C232%20in%202018.

[CIT0018] Jensen, M. B. 2003. The effects of feeding method, milk allowance and social factors on milk feeding behaviour and cross-sucking in group housed dairy calves. Appl. Anim. Behav. Sci. 80(3):191–206. doi:10.1016/S0168-1591(02)00216-2

[CIT0019] Jensen, M. B., K. S. Vestergaard, and C. C. Krohn. 1998. Play behaviour in dairy calves kept in pens: the effect of social contact and space allowance. Appl. Anim. Behav. Sci. 56(2–4):97–108. doi:10.1016/S0168-1591(97)00106-8

[CIT0020] Jorgenson, L. J., N. A. Jorgensen, D. J. Schingoethe, and M. J. Owens. 1970. Indoor versus outdoor calf rearing at three weaning ages. J. Dairy Sci. 53(6):813–816. doi:10.3168/jds.S0022-0302(70)86296-8

[CIT0021] Krohn, C. C., J. G. Jago, and X. Boivin. 2001. The effect of early handling on the socialisation of young calves to humans. Appl. Anim. Behav. Sci. 74(2):121–133. doi:10.1016/S0168-1591(01)00161-7

[CIT0022] Kung, L. Jr, S. Demarco, L. N. Siebenson, E. Joyner, G. F. Haenlein, and R. M. Morris. 1997. An evaluation of two management systems for rearing calves fed milk replacer. J. Dairy Sci. 80(10):2529–2533. doi:10.3168/jds.S0022-0302(97)76206-4 9361225

[CIT0023] Lago, A., S. M. McGuirk, T. B. Bennett, N. B. Cook, and K. V. Nordlund. 2006. Calf respiratory disease and pen microenvironments in naturally ventilated calf barns in winter. J. Dairy Sci. 89:4014–4025. doi:10.3168/jds.S0022-0302(06)72445-6 16960078

[CIT0024] Lauber, M. C. Y., P. H. Hemsworth, and J. L. Barnett. 2006. The effects of age and experience on behavioural development in dairy calves. Appl. Anim. Behav. Sci. 99(1–2):41–52. doi:10.1016/j.applanim.2005.10.009

[CIT0025] Lorenz, I., B. Earley, J. Gilmore, I. Hogan, E. Kennedy, and S. J. More. 2011. Calf health from birth to weaning. III. Housing and management of calf pneumonia. Ir. Vet. J. 64:14. doi:10.1186/2046-0481-64-14 22018053 PMC3220626

[CIT0026] Lundell, A. 2015. Advantages and disadvantages with outdoor hutches as housing system for calves and their future effect on the replacement heifer. Swedish University of Agricultural Sciences Faculty of Veterinary Medicine and Animal Science; p. 1–11. [bachelor thesis].

[CIT0027] Lv, J., X. W. Zhao, H. Su, Z. P. Wang, C. Wang, J. H. Li, X. Li, R. X. Zhang, and J. Bao. 2021. Effects of group size on the behaviour, heart rate, immunity, and growth of Holstein dairy calves. Appl. Anim. Behav. Sci. 241:105378. doi:10.1016/j.applanim.2021.105378Get

[CIT0028] Mahendran, S. A., D. C. Wathes, R. E. Booth, and N. Blackie. 2021. The health and behavioural effects of individual versus pair housing of calves at different ages on a UK commercial dairy farm. Animals 11(3):612. doi:10.3390/ani11030612 33652725 PMC7996845

[CIT0029] Marcé, C., R. Guatteo, N. Bareille, and C. Fourichon. 2010. Dairy calf housing systems across Europe and risk for calf infectious diseases. Animal 4:1588–1596. doi:10.1017/S1751731110000650. 22444707

[CIT0030] Medrano-Galarza, C., S. J. LeBlanc, T. J. DeVries, A. Jones-Bitton, J. Rushen, A. M. de Passillé, M. I. Endres, and D. B. Haley. 2018. Effect of age of introduction to an automated milk feeder on calf learning and performance and labor requirements. J. Dairy Sci. 101:9371–9384. doi:10.3168/jds.2018-14390 30055924

[CIT0031] Medrano-Galarza, C., S. J. LeBlanc, A. Jones-Bitton, T. J. DeVries, J. Rushen, A. M. de Passillé, and D. B. Haley. 2017. Producer perceptions of manual and automated milk feeding systems for dairy calves in Canada. Can. J. Anim. Sci. 98(2):250–259. doi:10.3168/jds.2016-12273

[CIT0032] Miller-Cushon, E. K., R. Bergeron, K. E. Leslie, G. J. Mason, and T. J. DeVries. 2014. Competition during the milk-feeding stage influences the development of feeding behavior of pair-housed dairy calves. J. Dairy Sci. 97:6450–6462. doi:10.3168/jds.2014-8065 25108872

[CIT0033] Miller-Cushon, E. K., and T. J. DeVries. 2015. Invited review: development and expression of dairy calf feeding behaviour. Can J. Anim. Sci. 95(3):341–350. doi:10.4141/cjas-2014-163

[CIT0034] Mogensen, L., C. C. Krohn, J. T. Sørensen, J. Hindhede, and L. H. Nielsen. 1997. Association between resting behaviour and live weight gain in dairy heifers housed in pens with different space allowance and floor type. Appl. Anim. Behav. Sci. 55(1–2):11–19. doi:10.1016/S0168-1591(97)00041-5

[CIT0035] Nordlund, K. V. 2008. Practical considerations for ventilating calf barns in winter. Vet. Clin. North Am. Food Anim. Pract. 24:41–54. doi:10.1016/j.cvfa.2007.10.006 18299031

[CIT0036] O’Brien, B., M. D. Gleeson, D.J. Ruane, J. Kinsella, and M. K. O’Donovan. 2007. New knowledge of facilities and practises on Irish dairy farms—fundamental requirements for effective extension. In: Proceedings of Association for international Agricultural and Extension Education; p. 270–279.

[CIT0037] Shalloo, L., A. Cromie, and N. McHugh. 2014. Effect of fertility on the economics of pasture-based dairy systems. Animal 8(Suppl 1):222–231. doi:10.1017/S1751731114000615 24679449

[CIT0038] Sinnott, A. M., E. Kennedy, and E. A. M. Bokkers. 2021. The effects of manual and automated milk feeding methods on group-housed calf health, behaviour, growth and labour. Livest Sci. 244:104–343. doi:10.1016/j.livsci.2020

[CIT0039] Sutherland, M. A., G. L. Lowe, F. J. Huddart, J. R. Waas, and M. Stewart. 2018. Measurement of dairy calf behavior prior to onset of clinical disease and in response to disbudding using automated calf feeders and accelerometers. J. Dairy Sci. 101:8208–8216. doi:10.3168/jds.2017-14207 29908799 PMC7094384

[CIT0040] Swanson, E. W., and J. D. Harris Jr. 1958. Development of rumination in the young calf. J. Dairy Sci. 41(12):1768–1776. doi:10.3168/jds.S0022-0302(58)91161-5

[CIT0041] Tapkı, İ., A. Şahin, and A. G. Önal. 2006. Effect of space allowance on behaviour of newborn milk-fed dairy calves. Appl. Anim. Behav. Sci. 99(1–2):12–20. doi:10.1016/j.applanim.2005.09.006.

[CIT0042] Teagasc. 2017. Individual and group housing. In: Teagasc, editor. Teagasc calf rearing manual. Ireland: Teagasc; p. 85–88.

[CIT0043] Van Os, J., S. Adcock, J. Costa, C. Halbach, T. Kohlman, E. Miller-Cushon, T. Ollivett, D. Sockett, and S. Stuttgen. 2021. Two heads are better than one: a starter guide to pairing dairy calves: hygiene practices. [accessed July 14, 2021]. https://animalwelfare.cals.wisc.edu/wp-content/uploads/sites/243/2021/02/03-Hygiene-practices.pdf.

[CIT0044] Webster, A. J., C. Saville, B. M. Church, A. Gnanasakthy, and R. Moss. 1985. The effect of different rearing systems on the development of calf behaviour. Br. Vet. J. 141:249–264. doi:10.1016/0007-1935(85)90061-2 4005517

[CIT0045] Whalin, L., D. M. Weary, and M. A. G. von Keyserlingk. 2018. Short communication: pair housing dairy calves in modified calf hutches. J. Dairy Sci. 101:5428–5433. doi:10.3168/jds.2017-14361 29605333

[CIT0046] Wójcik, J., R. Pilarczyk, A. Bilska, O. Weiher, and P. Sanftleben. 2013. Performance and health of group-housed calves kept in igloo calf hutches and calf barn. Pak. Vet. J. 33(2):175–178. ISSN: 0253-8318.

