# Supplementary material for: A comparison of indoor and outdoor calf housing systems using automated and manual feeding methods and their effect on calf health, behavior, growth, and labor
Source: J Anim Sci. 2022 Mar 15;100(4):skac079. doi: 10.1093/jas/skac079 (PMC9037364; doi:10.1093/jas/skac079)
Supplement: skac079_suppl_Supplementary_Files [file skac079_suppl_Supplementary_Files.docx]

**Supplementary Files**

| Age (weeks) | **0.5** | **1** | **1.5** | **2** | **2.5** | **3** | **3.5** | **4** | **4.5** | **5** | **5.5** | **6** | **6.5** | **7** | **7.5** | **8** |
| --- | --- | --- | --- | --- | --- | --- | --- | --- | --- | --- | --- | --- | --- | --- | --- | --- |
| Milk Quantity (L/day) | 6.0 | 6.0 | 6.0 | 6.0 | 6.5 | 7.0 | 7.5 | 8.0 | 8.0 | 8.0 | 7.0 | 6.0 | 5.0 | 4.0 | 3.0 | 2.0 |

**Supplementary File 1.** Individual calf milk feeding plan indicating calf milk quantity offered per day (L/day) relative to the age of the calf (in weeks).

**Supplementary File 2.** Definition of and scoring levels used in the health scoring system adapted by Sinnott et al (2021) from Teagasc (Sayers et al., 2016) and Barry et al (2019) to assess dairy calf health within the experiment.

| **Indicator** | **Definition** | **Scoring** | **Score Levels** |
| --- | --- | --- | --- |
| ***Demeanour*** | Combined evaluation of behaviour and responsiveness | 4-point scale of 0 to 3 | 0 = Bright, alert, responsive  1 = Dull, possibly depressed, less responsive  2 = Dull, markedly depressed, markedly unresponsive  3=Unresponsive to any stimulus |
| ***Nasal Discharge*** | Presence of any mucous discharge from the nasal passage | 4-point scale of 0 to 3 | 0 = clear, discharge free  1 = Small amount of cloudy mucous visible  2 = medium amount of bilateral muscous discharge  3 = excessive bilateral mucous discharge |
| ***Ocular Discharge*** | Position, appearance and presence of ocular discharge | 4-point scale of 0 to 3 | 0 = Bright, pronounced  1 = Slightly dull, presence of discharge  2 = Slightly dull, small amount of visible discharge |
|  |  |  | 3 = Dull, sunken, excessive amount of visible discharge |
| ***Ear Position*** | Positioning and activity of ears | 4-point scale of 0 to 3 | 0 = Alert and mobile  1 = Slightly drooped  2 = Drooped  3 = Drooped and limp |
| ***Cough*** | Presence of a cough, increased respiratory rate | 4-point scale of 0 to 3 | 0 = Normal Breathing  1 = Spontaneous coughing  2 = Intermittent coughing  3 = Continuous cough, increased respiration |
| ***Hydration*** | Appearance of calf eyes in relation to hydration levels | 4-point scale of 0 to 3 | 0 = Clear bright eyes  1 = Eyes slightly sunken  2 = Eyes sunken  3 = Eyes markedly sunken |
| ***Mobility*** | Ability to stand unassisted and move freely | 4-point scale of 0 to 3 | 0 = Stands unassisted, actively mobile  1 = Slow to stand, limited mobility  2 = struggles to stand, limited mobility  3 = Assistance required to stand, no mobility |
| ***Interest in Surroundings*** | Willingness to interact with observer | 2-point scale of 0 to 1 | 0 = Interactive when approached  1 = Uninterested when approached |
| ***Faecal Hygiene*** | Cleanliness of calf tail area and hindquarters | 4-point scale of 0 to 3 | 0 = completely clean hind quarters with no faecal matter  1 = slight faecal matter around hind quarters  2 = heavier faecal matter around hind quarter  3 = extremely dirty hind quarters and tail |

**Supplementary File 3.** Ethogram adapted by Sinnott et al (2021) from Barry et al. (2019), which categorises and defines various behaviours, used for behavioural observations

| **Category of behaviour** | **Behaviour** | **Definition** |
| --- | --- | --- |
| *Posture* | Standing | Calf is in a static upright standing position with weight placed on all four legs |
|  | Lying | Calf is resting either sternally or laterally with all four legs hunched close to body either awake or asleep. |
| *General* | Walking | Calf is actively moving from one point in the pen to another in an active walking motion |
|  | Not visible | Behaviour of the calf is not visible |
|  | Defecating/Urinating | Calf defecates or urinates |
| *Feeding Behaviour* | Drinking water | Calf is drinking water |
|  | Eating | Calf eats concentrates or roughage, or other solid feed (proximity of head to feed) |
| Comfort behaviour | Scratching/Rubbing/Stretching | Calf scratches itself with one of their legs (generally hind legs). Calf rubs itself on pen structure. Calf stretches itself. |
| *Abnormal behaviour* | Tongue playing/rolling | Calf makes repeated movements with its tongue inside or outside its mouth |
|  | Urine drinking / oral manipulate prepuce / cross sucking | Calf drinks the urine of another calf. Calf attempts to suck the naval area of another calf. Calf attempts to suck any body part of another calf. |
|  | Orally manipulating pen structure | Calf licks, nibbles, sucks, or bites at the pen structure (barriers, walls, buckets, troughs etc.) |
| *Play behaviour* | Play behaviour/ Mounting/ Head butting | Calf runs, jumps, changes direction suddenly, bucks, kicks hind legs, twists or rotates body. Calf mounts, or attempts to mount, a pen mate. Calf is engaged in head to head pushing with another calf. |
| *Social behaviour* | Social interaction | Calf licks another calf in the same area multiple times. Calf nudges another calf with its nose |

**Supplementary File 4.** Catalogue of definitions and cues adapted from Sinnott et al. (2021) used during labour evaluations to differentiate between tasks involved with indoor automatic feeding systems (IN_AUTO), indoor manual feeding systems (IN_MAN), outdoor group and individual hutches (OUT_G_HUTCH and OUT_I_HUTCH, respectively).

| Task | IN_AUTO | IN_MAN | OUT_G_HUTCH | OUT_I_HUTCH |
| --- | --- | --- | --- | --- |
| Milk Preparation | SW start when milk replacer bag opened. Hopper cover removed. Bag emptied into the hopper. SW stop when the hopper cover was replaced. | SW started when water entered first bucket. SW stopped when the last bucket of milk was made (mixed). | SW started when water entered first bucket. SW stopped when the last bucket of milk was made (mixed). | SW started when water entered first bucket. SW stopped when the last bucket of milk was made (mixed). |
| Transport | NA | NA | SW starts when first bucket lifted to be brought to form of transport. SW stops when housing destination is reached (includes to and from destination). | SW starts when first bucket lifted to be brought to form of transport. SW stops when housing destination is reached (includes to and from destination). |
| Feeding/Feeding Inspection* | SW started when hand-held automatic feeding monitor attached to feeder was inspected for calf drinking history. SW finished when all calves information had been checked. | SW starts when first bucket of milk was poured into feeders. Each calf inspected for obvious signs of ill health, they had a good demeanour, could stand and walk, and were consuming milk. SW stopped when last calf was given health check. | SW starts when first bucket of milk was poured into feeders. Each calf inspected for obvious signs of ill health, they had a good demeanour, could stand and walk, and were consuming milk. SW stopped when last calf was given health check. | SW starts when first bucket of milk was poured into individual feeders. Each calf inspected for obvious signs of ill health, they had a good demeanour, could stand and walk, and were consuming milk. SW stopped when last calf was given health check. |

| Cleaning Pen° | SW started when entered pen. Water, detergent and disinfectant was used to clean feeding area surfaces. SW finished when pen was clean. | SW started when entered pen. Water, detergent and disinfectant was used to clean feeding area surfaces. SW finished when pen was clean. | SW started when entered pen. Water, detergent and disinfectant was used to clean feeding area surfaces. SW finished when pen was clean. | | SW started when entered pen. Water, detergent and disinfectant was used to clean feeding area surfaces. SW finished when pen was clean. |
| --- | --- | --- | --- | --- | --- |
| Cleaning Equipment | SW starts when begin washing housing specific feeders. SW stops when respective feeders are cleaned | SW starts when begin washing housing specific feeders. SW stops when respective feeders are cleaned | SW starts when begin washing housing specific feeders. SW stops when respective feeders are cleaned | SW starts when begin washing housing specific feeders. SW stops when respective feeders are cleaned | |
| Health Check | SW starts when enter pen to inspect calf health. Each calf inspected for obvious signs of ill health, they had a good demeanour, could stand and walk .SW stops when last calf health is inspected | NA  *see feeding inspection | NA  *see feeding inspection | NA  *see feeding inspection | |

° excluding removal of soiled bedding, disinfection of surfaces and rebedding
